# Design and Experimental Study of a Tetragonal Rotor Pump Based on Wankel Geometry

**DOI:** 10.3390/s22176608

**Published:** 2022-09-01

**Authors:** Zhenzhen Gui, Xiaosi Zhou, Yaohua Zeng, Fan Zhang, Yuxuan Huo, Weirong Zhang, Mingdong Ma, Xi Huang, Jianhui Zhang

**Affiliations:** School of Mechanical and Electrical Engineering, Guangzhou University, Guangzhou 510006, China

**Keywords:** wankel pump, tetragonal, shape factor

## Abstract

Wankel pump designs have not been fully established, with existing designs limited to bicornous rotor pumps and triangular rotor pumps. Here, on the basis of Wankel geometry, we present a tetragonal rotor pump with a three-lobe epicycloid and its conjugate envelope as chamber and rotor profile. First, the design method and basic working principle of the pump are introduced. Four groups of prototypes with different shape factors were manufactured, and their flow and pressure characteristics were experimentally studied. Numerical study showed that the flow rate irregularity of the pump is lower than that of existing Wankel pumps. Finally, the feasibility the pump for mixing applications was verified by a flow field observation experiment. The work in this paper provides a new type of rotary displacement pump design, representing an study of reverse application of a Wankel engine structure.

## 1. Introduction

Wankel engines [1,2,3,4,5] are rotary positive-displacement machines. In a working cycle, the multichamber structure can independently generate multivolume periodic changes. Wankel engines have good capacity characteristics and can generate high specific power and high vacuum and are therefore used in positive-displacement compressors [6,7] and expanders [8,9,10,11], especially as rotary positive-displacement pumps, not only retaining the structural advantages of Wankel engines but also exerting the advantages of fewer parts, small size, minimal vibration, and compact structure [12,13,14,15,16,17,18]. Therefore, Wankel pumps are applied in the fields of medical treatment, industry, water and hydropower, etc.

The power of Wankel engines comes from the driving force generated by the explosion of chemical substances, which acts on the rotor to drive the rotating shaft connected to produce rotary motion. Reverse application of Wankel engines means that the power that produces the volume change comes from outside, and the volume change is used to push the fluid to do work. Existing rotary positive-displacement pump designs for reverse applications of Wankel engines are limited to bicornous rotor pumps (with a two-lobe inner envelope) [12,13,14,15,16,17,18,19,20,21] and triangular rotor pumps (with a three-lobe inner envelope) [22,23,24]. Monties et al. (1990) first proposed the application of a bicornous rotor pump based on Wankel geometry to an artificial heart [14]. Such pumps are capable of self-priming and do not require a valve structure, with a continuous and pulsating flow. Another study by Monties et al. (1994) showed that controlling the gap between the rotor and the stator within a certain range can limit the generation of turbulence, resulting in a shear rate lower than the hemolysis threshold [13]. Monties et al. (1996) designed a seal gear structure using ultra-precision machining by polishing the components of the angular rotor pump and proposed that if the bearing sealing problem can be solved, an angular rotor pump can be used as a permanent left ventricular assist device [17]. The same authors (2010) studied the materials for the manufacture of bicornous rotor pumps and confirmed that Ti_6_Al_4_V has good biocompatibility [16]. This research group [13,14,15,16,17] has made continuous efforts toward the development of an angular rotor pump as an artificial heart, and their work shows that angular rotor pumps exhibit high pulsation and are only suitable for low-speed and small-scale applications, such as artificial hearts. Wan et al. (2014) simulated the internal flow field of a bicornous rotor pump, finding that such a pump has abundant three-dimensional vortex structures, even in the laminar flow state, and first proposed the use a bicornous rotor pump as a microreactor and a micromixer [21]. Lau et al. (2016) verified the feasibility of mixing two highly viscous fluids with a bicornous rotor pump in a visualization experiment [19]. In the same year (2016), Lim et al. designed a special rotating shaft whereby the rotating motion of the rotor was guided by the linear motion of the rotating shaft in the inner groove of the rotor, which solves the problem of inconvenient installation of the rotating shaft in a long and narrow bicornous rotor [20]. Wan et al. (2017) developed a single-stage, two-stage (parallel), double-cylinder bicornous rotor pump and highlighted the potential and challenges associated with the development of a bicornous rotor pump as a mixer [12]. The development of triangular rotor pumps has mainly been carried out in China; Li et al. (2018) compared the performance of a triangular rotor grouting pump with that of existing grouting pumps (such as diaphragm pumps and plunger pumps) and found that the grouting efficiency of triangular rotor pumps is 10 times higher than that of diaphragm pumps and that the flow rate is significantly higher than that of plunger pumps, maintaining comparable high pressure [22]. The same authors (2019) analyzed the sealing structure of a rotary piston pump and found that working pressure, crankshaft speed and the friction coefficient between the sealing strip and the inner wall of the cylinder are factors that affect the wear of the sealing strip [24].

In existing studies, due to the large difference between the maximum chamber volume and the minimum chamber volume, the flow rate irregularity of bicornous rotor pumps is highly irregular, and the installation of the rotation axis in the rotor is not convenient, requiring a specially designed rotation axis to guide the movement, limiting their application to mixers or artificial hearts. Triangular rotor pumps can achieve good performance and are suitable for grouting, although with high flow rate irregularity. Experience obtained to date is based on bicornous rotor pumps and triangular rotor pumps, and the design parameters of Wankel pumps have not been fully established. In this paper, based on the inspiration of Wankel engine structure and on the basis of the existing Wankel pumps, we propose a tetragonal rotor pump with a three-lobe epicycloid and its conjugate envelope as the profile of the chamber and rotor. The proposed design is a rotary displacement pump with lower flow rate irregularity than bicornous rotor pumps and triangular rotor pumps, which may be more advantageous for some applications. First, the mathematical description of a quadrilateral rotor pump was established. Then, kinematic analysis of the pump was carried out to explore the influence of different shape factors on the motion and flow characteristics of the pump. Finally, the feasibility applying such a structure to a pump was experimentally verified, and the flow characteristics and pressure characteristics of the pump were obtained. This research is an exploratory and tentative extension of reverse application of the Wankel engine structure.

## 2. Structure and Working Principle of the TRP

**Figure 1:**
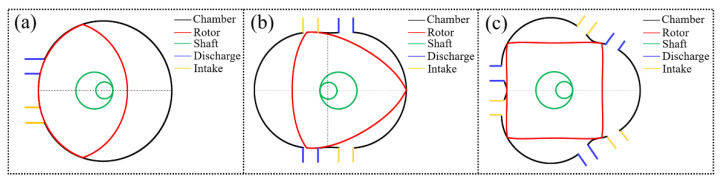
Structural diagram of an existing Wankel pump and the TRP proposed in this paper. (**a**) Bicornous rotor pump; (**b**) triangular rotor pump; (**c**) tetragonal rotor pump.

In a Wankel engine, each working cycle consists of four working strokes, namely intake, compression, work, and exhaust. When used as a pump, the intake and power strokes of the engine are originally occur as a suction process, and the original compression and exhaust strokes are performed as a discharge process. Therefore, when the motion of the engine is used for a rotary positive-displacement pump, a set of suction and discharge ports are added to the chamber, and the TRP’s structure has one more set of chambers than that of a triangular rotor pump. A set of suction ports and discharge ports therefore must be added to the chamber. A bicornous rotor pump’s structure has one fewer set of chambers than a triangular rotor pump, and a set of suction ports and discharge ports is correspondingly removed, as shown in Figure 1.

**Figure 2:**
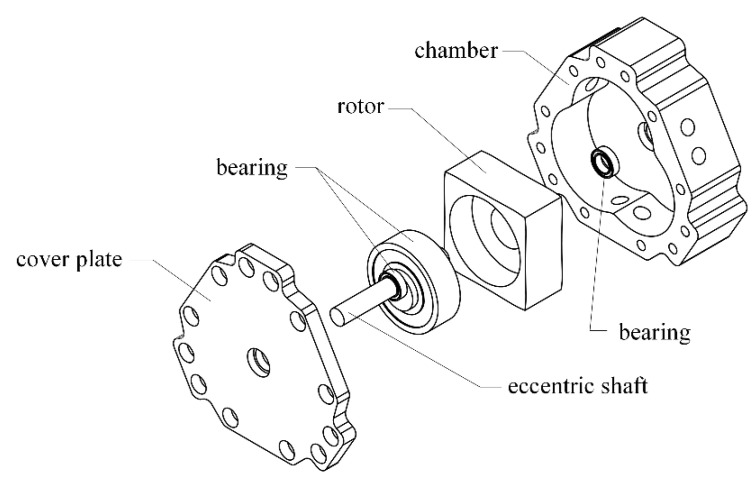
The exploded view of the TRP.

**Figure 3:**
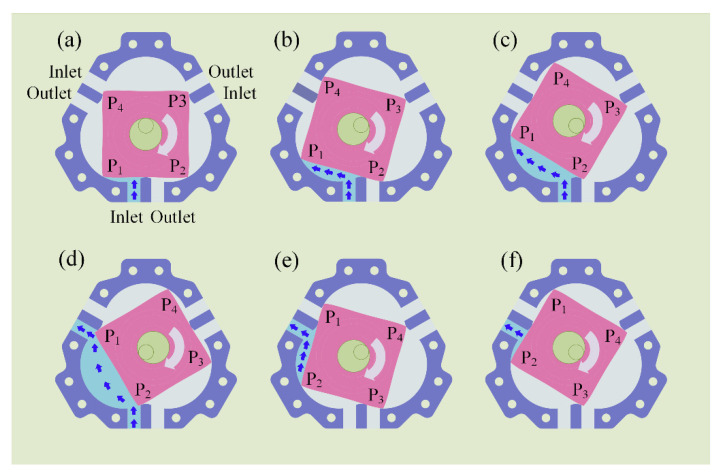
Working principle of TRP. (**a**) Suction stroke begins; (**b**,**c**) transition phase of suction stroke; (**d**) suction volume of the chamber reaches the maximum; (**e**) chamber enters the scheduling stage; (**f**) schedule ends.

An expanded view of the TRP is shown in Figure 2. For a single-cylinder TRP, the main components are the chamber, rotor, eccentric shaft, bearing, cover plate, etc. The crankshaft consists main journal and connecting journal. The main journal and the chamber are coaxial, and the connecting journal and the rotor center are coaxial. The four apexes of the rotor are in contact with the inner wall of the chamber so that there are four independent chambers between the rotor and the chamber (we define a rotor surface corresponding to a chamber). As the rotor rotates, the volume of the chamber changes periodically so as to complete the suction and discharge work. The working principle of the TRP with a complete working process of the rotor surface ($P_{1}P_{2}$,) is shown in Figure 3. At the position shown in Figure 1a, the volume of the chamber corresponding to the rotor face ($P_{1}P_{2}$) is the lowest, and the suction stroke has just started. As the shaft rotates, liquid is continuously sucked into the chamber, as show in Figure 1b,c. When the rotating shaft rotates to the position shown in Figure 1d, the suction volume of the chamber reaches the maximum, which is the separation point between the suction stroke and the discharge stroke. When the shaft continues to rotate, the chamber enters the scheduling stage, as shown in Figure 1e. When the rotor rotates to the position shown in Figure 1f, the schedule ends. The rotor changes from the position shown in Figure 1a to the position shown in Figure 1f, and the chamber corresponding to the rotor surface ($P_{1}P_{2}$) performs a complete suction stroke. Because the structure is centrosymmetric, the rotor faces ($P_{1}P_{2}$) repeat the process (a)–(f) after they have rotated through the position shown in Figure 1f, and the adjacent rotor faces ($P_{2}P_{3}$, $P_{3}P_{4}$, $P_{4}P_{1}$) are subject the same working conditions, with a difference in the time at which they complete the suction schedule.

## 3. Mathematical Description of the Pump Geometry

### 3.1. Mathematical Equations of Chamber Profile

**Figure 4:**
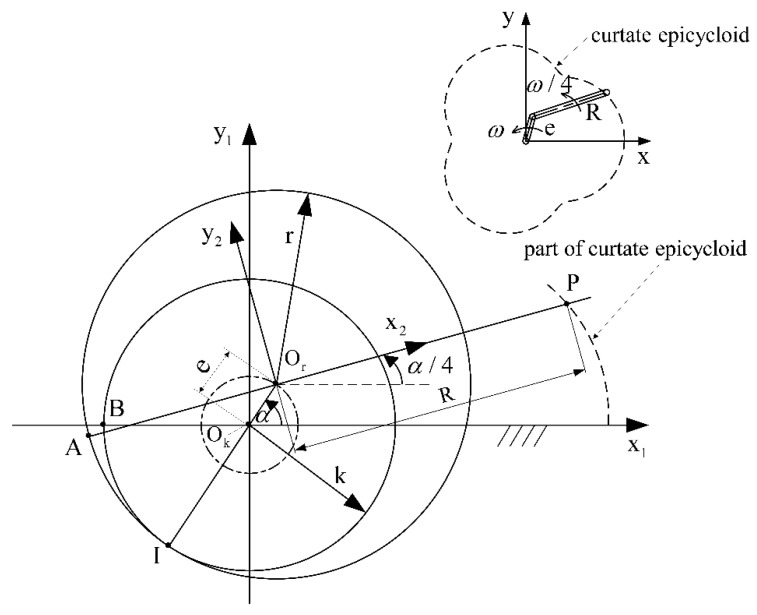
Generation schematic for the generating chamber profile.

As shown in Figure 4, a rolling moving circle ($O_{r}$) rolls along the outer circumference of another fixed circle ($O_{k}$) without sliding, with a radius ratio of 4:3. A fixed coordinate system ($x_{1}O_{k}y_{1}$) is established on the fixed circle ($O_{k}$), and the other rotating coordinate system ($x_{2}O_{r}y_{2}$) is established on the rolling circle ($O_{r}$). The origin of the two coordinate systems coincides with the center of the circle. The two circles start tangent to point B, i.e., point A and point B coincide. Figure 4 shows the position of the moving circle when it rolls over $\hat{\mathrm{AI}}$. The trajectory depicted by point P fixedly connected by the moving circle is the chamber profile. Mathematically, this type of line is called an epicycloid [25]. It is equivalent to the trajectories generated by the end of the connecting rod when the crank connecting the rod structure with side lengths *e* and *R* rotates in the same direction at the angular velocities of ω and ω/4, respectively. Based on the geometric relationship shown in Figure 1, the expression of the chamber profile can be obtained as:

(1) $$\{\begin{array}{l} x=e\cos\alpha +R\cos\frac{\alpha }{4}\\ y=e\sin\alpha +R\sin\frac{\alpha }{4} \end{array}$$

where *e* is the eccentricity, *R* is the generating radius, and *α* is the angular displacement of the eccentric shaft. *α* takes [0, 8π] to obtain the complete chamber profile.

### 3.2. Mathematical Equations of Rotor Profile

**Figure 5:**
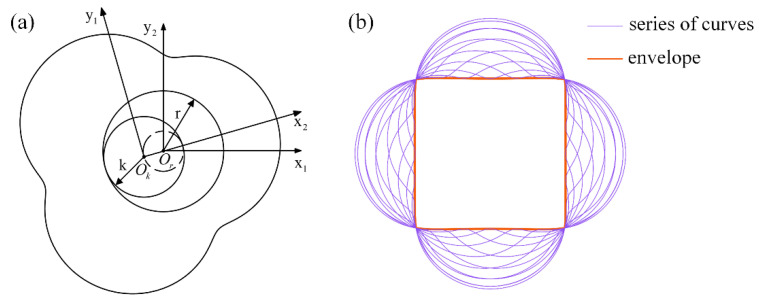
Generation principle of the rotor profile. (**a**) Generation mode; (**b**) generated series of curves and envelopes.

Contrary to the above method of generating the chamber profile, circle $O_{k}$ is used as a rolling circle, circle $O_{r}$ is a fixed circle, and the chamber profile is rolled around the fixed circle ($O_{r}$) with the rolling circle ($O_{k}$), as shown in Figure 5a, generating a series of curves, as shown in Figure 5b. The inner edge boundary line of this family of curves is called the conjugate envelope, which is the rotor profile [26], and the profile equation is described by expression (2). Therefore, if circle $O_{r}$ is a rolling circle and $O_{k}$ is a fixed circle, the generated inner envelope will roll along with circle $O_{r}$ around the fixed circle ($O_{k}$), meaning that the rotor can rotate in the chamber without interference.

(2) $$\{\begin{array}{c} X=R\cos(2ev)-\frac{4e^{2}}{R}\sin(8ev)\sin(2ev)\pm 2e\cos(4ev)\cos(2ev)\sqrt{1-\frac{16e^{2}}{R^{2}}\sin^{2}(4ev)}\\ Y=R\sin(2ev)+\frac{4e^{2}}{R}\sin(8ev)\cos(2ev)\pm 2e\cos(4ev)\sin(2ev)\sqrt{1-\frac{16e^{2}}{R^{2}}\sin^{2}(4ev)} \end{array}$$

Expression (2) represents two equation systems (both positive and negative), and the value range of *v* is [π/8*e*, 3π/8*e*] and [5π/8*e*, 7π/8*e*].

### 3.3. Shape Factor K

The chamber profile and rotor profile have two independent parameters, *e* and *R*, and their ratio, *R*/*e*, is called shape factor *K*. Through expressions (1) and (2), when the distance of eccentricity (*e*) and the generating radius (*R*) are determined, the chamber profile and the rotor profile can also be determined. When *e* and *R* are reduced or enlarged in the same proportion, the profile lines are also enlarged or reduced accordingly, and their shapes are similar. By dividing the left and right sides of the equation of expression (1) by *e* at the same time, a new expression can be obtained. This expression is only related to dimensionless value *K*. Similarly, the left and right sides of the equation of expression (2) can be divided by *R* at the same time to obtain a new expression related only to dimensionless value 1/*K*.

**Figure 6:**
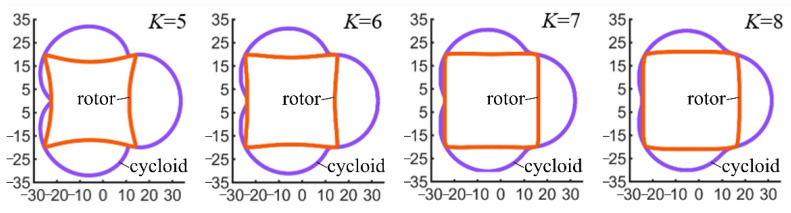
Cycloid and rotor profile with different shape factors.

Therefore, *K* is an important factor for designing pumps. In industrial applications, *K* range from 4 to 10, with the value of *K* most commonly falling between 6 and 8 [27]. When evaluating the influence of different rotor and chamber shape designs on the motion characteristics of a tetragonal rotor pump, in order to ensure that the overall size of the pump is consistent, we set the generating radius (*R*) as a constant, change the eccentricity, and select four sets of *K* values: 5, 6, 7, and 8. Table 1 shows the shape factors for the four cases. We compared the contours generated by different shape factors (*K*) in the design of a tetragonal rotor pump, as shown in Figure 6. With respect to the chamber profile, the larger the value of *K*, the larger the curvature radius of the recess in the curve and the smoother the curve transition. With respect to the rotor profile, the larger the *K* value, the more the line expands outward. The larger the corresponding *K* value, the lower the working chamber volume between the chamber and the rotor.

## 4. Kinematic Analysis of the TRP

### 4.1. Velocity Analysis of the TRP

In a reciprocating piston engine, the lifespan of the piston chamber and piston ring is estimated by the average moving speed of the piston. Likewise, the rotor apex speed is an important factor affecting the wear effect of the interface between the rotor and the chamber of the TRP. The coordinates determined by equation (1) also represent the instantaneous position of the rotor apex in contact with the housing. By taking the first-order time derivative of time *t*, the horizontal velocity and vertical velocity of the apex can be obtained:

(3) $$\{\begin{array}{l} v_{x}=\frac{dx}{dt}=-\omega e(\sin\alpha +\frac{1}{4}K\sin\frac{\alpha }{4})=-\omega R(\frac{1}{K}\sin\alpha +\frac{1}{4}\sin\frac{\alpha }{4})\\ v_{y}=\frac{dy}{dt}=\omega e(\sin\alpha +\frac{1}{4}K\cos\frac{\alpha }{4})=\omega R(\frac{1}{K}\cos\alpha +\frac{1}{4}\cos\frac{\alpha }{4}) \end{array}$$

where *ω* is the angular velocity of the eccentric shaft, which is a constant when the rotational speed is constant.

The apex circumferential velocity is described by the follow equation:

(4) $$v_{p}=\sqrt{{\left(\frac{dx}{dt}\right)}^{2}+{\left(\frac{dy}{dt}\right)}^{2}}=\frac{\omega e}{4}\sqrt{16+8K\cos\frac{3\alpha }{4}+K^{2}}=\frac{\omega R}{4}\sqrt{\frac{16}{K^{2}}+\frac{8}{K}\cos\frac{3\alpha }{4}+1}$$

Apex circumferential velocity number ($C_{1}$) is defined as a dimensionless group to eliminate the effect of different shaft rotational speeds and dimensions of the pump, which can be expressed as:

(5) $$C_{1}=\frac{v_{p}}{\omega R}=\frac{1}{4}\sqrt{\frac{16}{K^{2}}+\frac{8}{K}\cos\frac{3\alpha }{4}+1}$$

**Figure 7:**
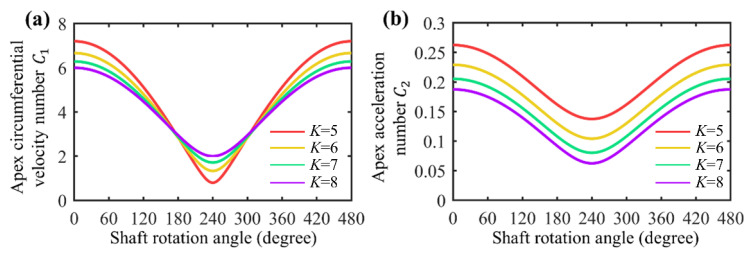
Kinematic characteristics with different *K* values. (**a**) Apex circumferential velocity number ($C_{1}$) versus *K*; (**b**) apex circumferential acceleration number ($C_{2}$ ) versus *K*.

Figure 7a shows the plot of apex circumferential velocity number ($C_{1}$) with respect to the shaft rotation angle for different shape factors. Decreasing the shape factor magnitude increases the maximum circumferential velocity and decreases the minimum circumferential velocity. The fluctuation of the curve is increased with lower *K* values. From the perspective of apex circumferential velocity, a larger shape factor is beneficial to reduce the interface wear between the rotor and the chamber.

### 4.2. Acceleration Analysis of TRP

By taking the second-order time derivative of equation (1), the horizontal acceleration and vertical acceleration of the apex can be obtained:

(6) $$\{\begin{array}{c} a_{x}=\frac{dv_{x}}{dt}=-\omega^{2}R(\frac{1}{K}\cos\alpha +\frac{1}{16}\cos\frac{\alpha }{4})\\ a_{y}=\frac{dv_{y}}{dt}=-\omega^{2}R(\frac{1}{K}\sin\alpha +\frac{1}{16}\sin\frac{\alpha }{4}) \end{array}$$

where *ω* is the angular velocity of the eccentric shaft, which is a constant when the rotational speed is constant.

The apex circumferential acceleration is described by the follow equation:

(7) $$a_{p}=\pm \sqrt{a_{x}^{2}+a_{y}^{2}}=\pm \omega^{2}R\sqrt{\frac{1}{256}+\frac{1}{K^{2}}+\frac{1}{8K}\cos\frac{3\alpha }{4}}$$

Apex circumferential acceleration number ($C_{2}$) is defined as a dimensionless group to eliminate the effect of different shaft rotational speeds and dimensions of the pump. The apex circumferential acceleration number ($C_{2}$) can be expressed as:

(8) $$C_{2}=\frac{a_{p}}{\omega^{2}R}=\sqrt{\frac{1}{256}+\frac{1}{K^{2}}+\frac{1}{8K}\cos\frac{3\alpha }{4}}$$

Figure 7b shows the plot of apex circumferential acceleration number ($C_{2}$) with respect to the shaft rotation angle for different shape factors. As the shape factor (*K*) decreases, the circumferential acceleration of the apex at the same rotation angle of the shaft increases, and a rotor with a shape factor of *K* = 5 has the largest maximum circumferential acceleration. From the perspective of circumferential acceleration, higher *K* values are conducive to reducing the interface wear between the rotor and the chamber.

## 5. Flow Rate of the TRP

### 5.1. Theoretical Flow Rate

**Figure 8:**
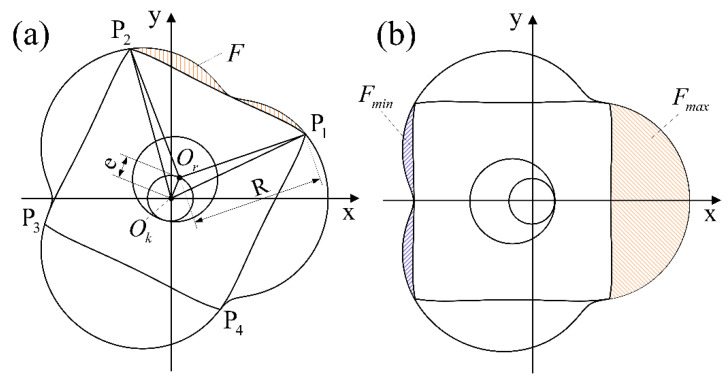
Chamber area varying with rotation angle. (**a**) Chamber area at any rotation angle; (**b**) the locations of the maximum and minimum chamber areas.

The rotor follows a path that keeps each of the four apexes of the rotor in contact with the chamber, creating four separate chamber volumes. When the rotational speed is constant, the volume of the chamber changes periodically, owing to which the volume change law of a single chamber is the same as that of adjacent chambers. The volume of a single chamber can be obtained by dividing the area, as shown in Figure 8a. $P_{1}$,$P_{2}$,$P_{3}$, and $P_{4}$ are the four apexes of the rotor, $O_{k}$ is the center of the chamber, and $O_{r}$ is the geometric center of the rotor. The chamber area (*F*) varies as the rotor rotates. Figure 8b shows the locations of the maximum and minimum chamber areas.

(9) $$\{\begin{array}{l} F_{1}=\pi (e^{2}+\frac{R^{2}}{4})-\frac{5\sqrt{2}}{6}Re\sin(\frac{3\alpha }{4}+\frac{\pi }{4})\\ F_{2}=\frac{1}{2}Re\sin(\frac{3\alpha }{4})\\ F_{3}=\frac{1}{2}Re\cos(\frac{3\alpha }{4})\\ F_{4}=(2e^{2}+R^{2})\frac{\pi }{4}-\frac{3Re}{2}\sqrt{1-\frac{16e^{2}}{R^{2}}}-(\frac{R^{2}}{8}+4e^{2})\sin^{-1}\frac{4e}{R} \end{array}$$

where $F_{1}$ is the area of curved sector $O_{k}P_{1}P_{2}$; $F_{2}$ is the area of triangle $O_{k}O_{r}P_{1}$; $F_{3}$ is the area of triangle $O_{k}O_{r}P_{2}$; and $F_{4}$ is the area of curved sector $O_{r}P_{1}P_{2}$, which accounts for a quarter of the rotor area.

The chamber area (*F*) can be calculated by $F=F_{1}-F_{2}-F_{3}-F_{4}$:

(10) $$F=-\frac{4\sqrt{2}Re}{3}\sin(\frac{3\alpha }{4}+\frac{\pi }{4})+\frac{e^{2}\pi }{2}+\frac{3Re}{2}\sqrt{1-{\left(\frac{4e}{R}\right)}^{2}}+(\frac{R^{2}}{8}+4e^{2})\sin^{-1}\frac{4e}{R}$$

The change in cavity area with the rotation angle can be described by a constant plus trigonometric function [28].

The volume of the chamber ($V_{c}$) can be calculated by multiplying right-hand side of Equation (10) by the thickness (*B*) of the rotor:

(11) $$V_{c}=FB$$

To study the relationship between the chamber volume and the shape factor (*K*), the chamber area divided by the square of the generating radius is defined as the chamber volume number ($C_{3}$) (dimensionless), which can be expressed as:

(12) $$C_{3}=\frac{F}{R^{2}}=-\frac{4\sqrt{2}}{3K}\sin(\frac{3\alpha }{4}+\frac{\pi }{4})+\frac{\pi }{2K^{2}}+\frac{3}{2K}\sqrt{1-\frac{16}{K^{2}}}+(\frac{1}{8}+\frac{4}{K^{2}})\sin^{-1}\frac{4}{K}$$

**Figure 9:**
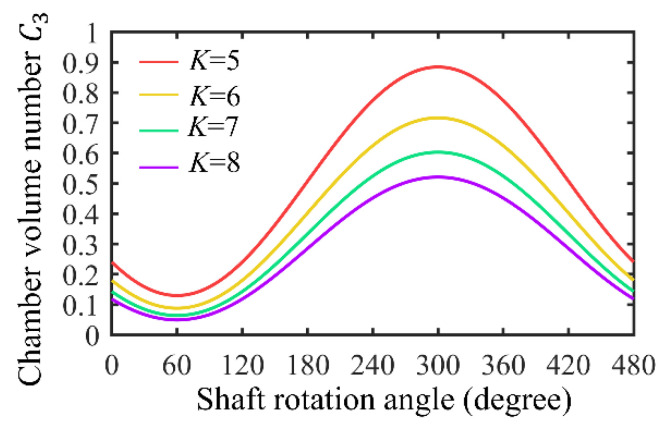
Effect of shape factor (*K*) on chamber volume number ($C_{3}$).

Figure 9 shows the chamber volume fluctuation for *K* from 5 to 8; the chamber volume varies sinusoidally with the rotation angle.

Similar to the definition of the displacement of a conventional piston engine [29], the displacement of the TRP (*V*) is the volume of the liquid discharged per revolution of the rotating shaft. As shown in Figure 2, the rotor changes from the position shown in process (a) to (f), the rotor surface ($P_{1}P_{2}$) rotates 120°, and the chamber corresponding to the rotor surface ($P_{1}P_{2}$) completes one suction stroke and one discharge stroke; equivalently, when the rotor rotates one circle, the chamber corresponding to rotor surface ($P_{1}P_{2}$) completes three suction strokes and three discharge strokes, and the rotor has four rotor surfaces, so when the rotating shaft rotates four times (the rotor rotates once), the TRP completes a total of twelve suction strokes and twelve discharge strokes. In other words, three suction strokes and three scheduling strokes are completed for each rotation of the shaft. The displacement (*V*) can be expressed as:

(13) $$V=3△FB=3(F_{max}-F_{min})B=8\sqrt{2}ReB=\frac{8\sqrt{2}R^{2}B}{K}$$

where *B* is the thickness of the rotor, $F_{max}$ is the maximum chamber area, and $F_{min}$ is the minimum chamber area.

Under the assumption that the fluid is incompressible and the four chambers are isolated from each other, the theoretical flow rate (*Q*) of the TRP can be expressed as:

(14) Q=nV

where *n* is the rotational speed of the shaft.

### 5.2. Specific Flow Rate

The specific flow rate (*i*) is defined as the ratio between the volume of fluid conveyed in one revolution of the rotor (displacement, *V*) and the volume occupied by the rotor assembly:

(15) $$i=\frac{V}{4F_{4}B}=\frac{8\sqrt{2}K}{\left[(2+K^{2})\pi -6\sqrt{K^{2}-16}-(\frac{K^{2}}{2}+16)\sin^{-1}\frac{4}{K}\right]B}$$

**Figure 10:**
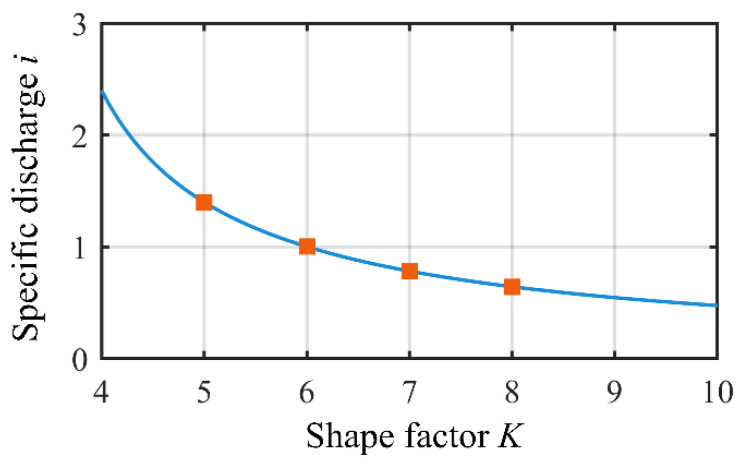
Effect of shape factor (*K*) on specific discharge (*i*).

Figure 10 shows the relationship between the specific flow rate (*i*) and the shape factor (*K*); as *K* increases, the specific flow rate (*i*) decreases. A low *K* value corresponds to a low rotor volume, as shown in Figure 6, with a high specific flow rate, i.e., the rotor per unit volume can pump more fluid.

### 5.3. Flow Rate Irregularity

Owing to their geometry, TRPs are not able to supply users with a constant flow rate. The decrease in chamber volume is equal to the increase in outlet medium. Considering the delivered fluid as incompressible, the instantaneous flow rate (*q*) delivered by the chamber can be obtained by differentiating the chamber volume ($V_{c}$) with respect to time:

(16) $$q=\frac{dV_{c}}{dt}=-\sqrt{2}\omega BRe\cos(\frac{3\alpha }{4}+\frac{\pi }{4})$$

A positive and negative value of the instantaneous flow rate indicates the direction of the flow. A negative instantaneous flow rate indicates instantaneous flow at the outlet. A positive instantaneous flow rate represents instantaneous flow at the inlet.

Because the chamber volume of the TPR changes sinusoidally, its flow pulsates periodically as the rotor rotates. The concept of flow rate irregularity (*σ*) is introduced to measure the magnitude of flow pulsation:

(17) $$\sigma =\frac{q_{max}-q_{min}}{Q}\%$$

where $q_{max}$ is the maximum instantaneous flow, $q_{min}$ is the minimum instantaneous flow, and *Q* is the theoretical flow rate.

By combining Equations (15)–(17), the flow rate irregularity ($\sigma_{T}$) of the TRP can be obtained:

(18) $$\sigma_{T}=\frac{\pi }{4}$$

The chamber volume of a triangular rotor pump [23,24,27,30] and a bicornous rotor pump [13,14,15,17] also changes sinusoidally with the rotating shaft, and the flow rate irregularity can be obtained by the same method:

(19) $$\{\begin{array}{c} \sigma_{t}=\frac{\pi }{3}\\ \sigma_{b}=\frac{\pi }{2} \end{array}$$

Here, $\sigma_{t}$ represents the flow rate irregularity of a triangular rotor pump, and $\sigma_{b}$ represents the flow rate irregularity of a bicornous rotor pump.

**Figure 11:**
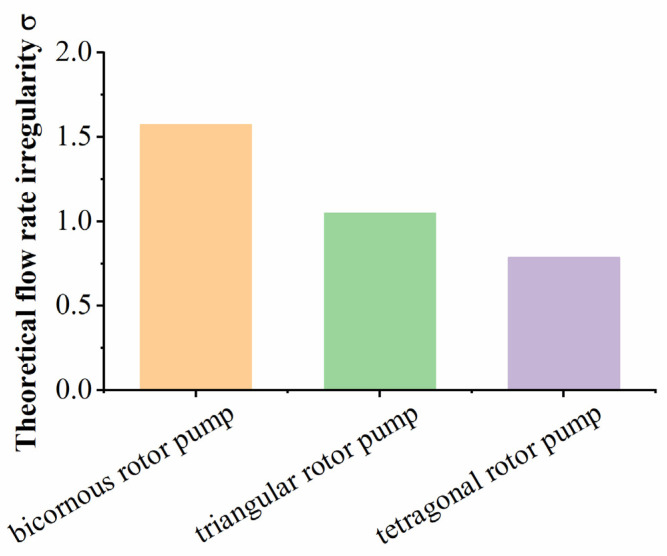
Comparison of flow rate irregularity of different Wankel pumps.

Equations (18) and (19) show that the theoretical flow rate irregularity of the same type of rotor pump is consistent and independent of the structural parameters (*R* and *e*) of the pump. The flow rate irregularity of a bicornous rotor pump is the highest of the three pump types, decreasing with increased in lobe number of the pump, as shown in Figure 11. The calculation results confirm the advantages of the TRP.

## 6. Experiments

### 6.1. Experimental Design

**Figure 12:**
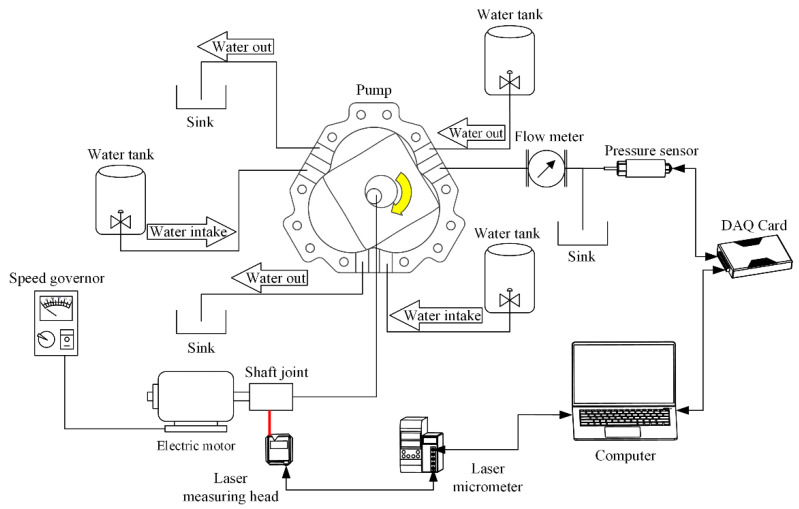
Schematic of the experimental setup (red line indicates the laser, and the yellow arrow indicates the direction of rotation).

In order to explore the influence of different shape factor designs on flow and pressure, we selected four shape factors to construct pump prototypes and tested them. The shape factors are shown in Table 1. After the shape factors are determined, the thickness of the rotor and the size and position of the ports (inlet and outlet) are also obtained. The specific structural parameters are shown in Table 2. We built a test system consisting of test pumps, a data collector, a DC geared motor, a governor, a laser displacement sensor, a pressure sensor (with an accuracy of 0.25%), a flowmeter, etc. A diagram of the system assembly diagram is shown in Figure 12. A high-speed camera was used to capture the flow field when the pump was working.

### 6.2. The Impact of Shape Factor on Flow Rate

We adjusted the motor speed (*n*) by with governor to be 160, 180, 200, 220, 240, 260, or 280 r/min and monitored the motor speed with a laser displacement sensor. Because the difference in flow rate between the three outlets was very small, we recorded the average flow rate for a single outlet in one minute.

**Figure 13:**
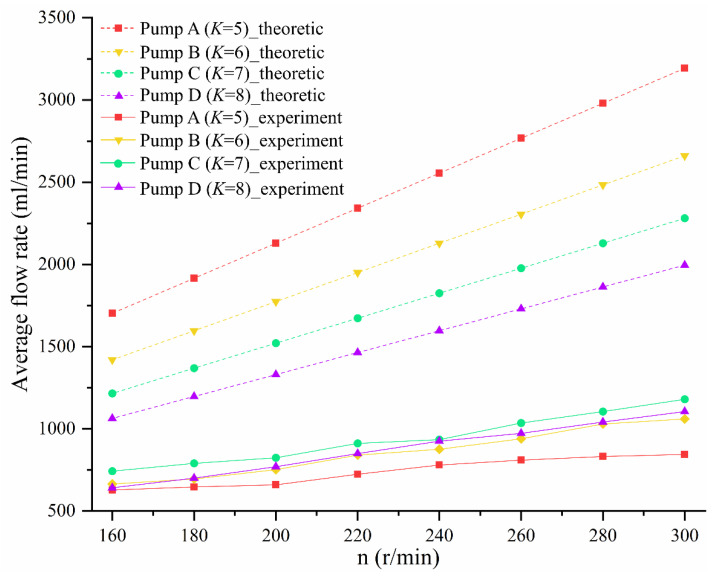
Change in average flow rate with speed.

**Figure 14:**
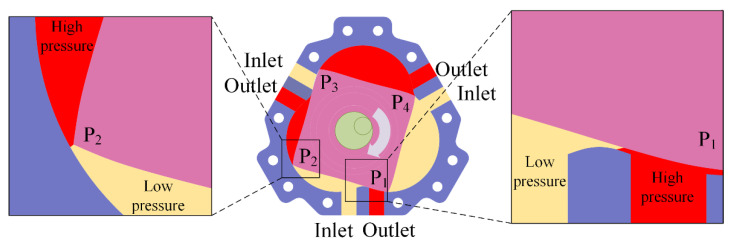
Clearance between the rotor and the inner wall of chamber.

Figure 13 shows the average flow rate of four groups of pumps with different *K* values in the speed range of 160~280 r/min. The dotted line is the theoretical average flow rate, and the solid line is the average experimentally measured flow rate. The average flow rates of the four groups of pumps with different *K* values increase with increased rotational speed, indicating that they are characteristics of positive-displacement pumps. One of the reasons for the difference between the actual flow rate and the theoretical flow rate is machining error. As shown in Figure 14, there is a gap between the rotor and the inner wall of the chamber, and the space between two adjacent chambers belongs to both the high-pressure area and the low-pressure area, resulting in leakage. Figure 14 shows that as the shaft rotates, the volume of the chamber corresponding to the rotor surface ($P_{1}P_{2}$) increases, and its vacuum degree also increases; the chamber is in a low-pressure state, and the adjacent chambers (the chambers corresponding to $P_{2}P_{3}$ and $P_{4}P_{1}$) are in a high-pressure state, so the high-pressure liquid in the adjacent chamber flows into the chamber corresponding to $P_{1}P_{2}$, resulting in leakage and reduced flow. The leakage can be confirmed by the flow field observation experiment described in Section 6.4. Figure 9 shows that the lower the *K* value, the greater the rate of change of the chamber volume with the rotation angle, that is, when the shaft rotates at the same angle, the lower the *K* value, the greater the vacuum generated by the pump, which sucks more fluid from the inlet. Furthermore, the amount of fluid flowing into adjacent chambers may also increase. Another possible reason for the difference between the actual flow and the theoretical flow is the geometric structure of the pump. There are differences between the rotor profile and the cavity profile of the pump depending on the *K* value, and the space between them also varies (see Figure 6), resulting in the varied flow resistance of the fluid flow. Under the influence of processing error and the flow resistance of the pump itself, pump A (*K* = 5) with the lowest *K* value has the lowest actual flow, whereas pump B (*K* = 6) and pump D (*K* = 8) have similar values, and the actual flow rate of pump C (*K* = 7) is the highest of the tested pumps. The result shows the difference between the actual flow rate curve and the theoretical flow rate curve.

### 6.3. The Impact of Shape Factor on Pressure

**Figure 15:**
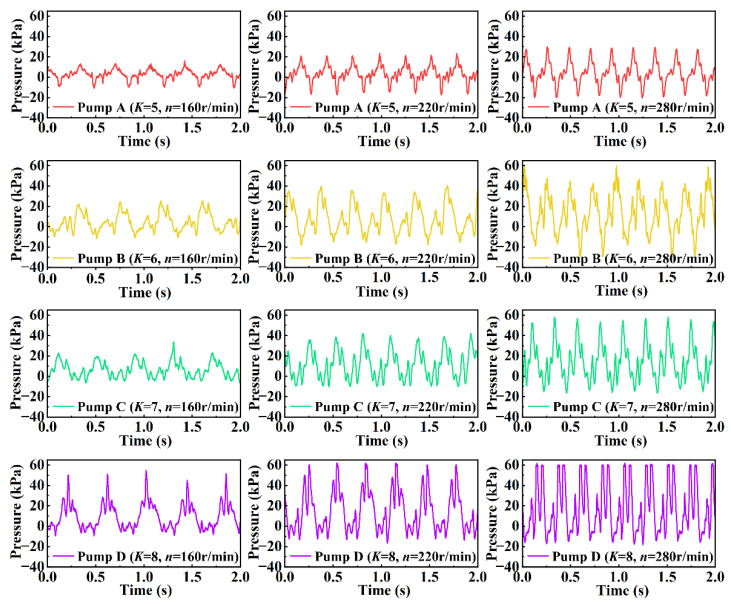
Pressure changes over time.

Figure 15 shows the curves of the pressure changes with time for four groups of pumps with different *K* values at rotational speeds of 160 r/min, 220 r/min, and 280 r/min. Comparing the pressure–time curves in the figure, the following conclusions can be drawn: For pumps with the same shape factor, the higher the rotational speed of the rotating shaft, the greater the frequency of pressure fluctuations and the greater the amplitude of pressure fluctuations because the rotor surface sweeps the outlet once, resulting in a pressure fluctuation, and the frequency of the fluctuation reflects the speed of the rotor. In addition, when the outlet area of the pump is constant, the higher the rotational speed of the rotating shaft, the faster the change rate of the volume of the pump chamber and the faster the delivery rate. The larger the kinetic energy of the liquid, the larger the amplitude of the pressure fluctuation; for pumps with different shape factors at the same speed, the larger the shape factor (*K*) value, the larger the amplitude of the outlet pressure of the pump. Under the condition of constant output power, the higher the *K* value, the lower the outlet flow rate, and the corresponding outlet pressure is also higher. The pressure–time curve does not steadily increase to the maximum value and then decrease; instead, before the curve rises to the maximum pressure value, there is a small fluctuation, followed by another small fluctuation after the curve rises to the maximum value. This occurs because the water inlet in the chamber is open at the beginning of the discharge stroke of the rotor, but the water outlet is not closed (the inlet and outlet open simultaneously in the chamber), and as the rotor rotates, the rotor surface covers the water inlet at the rear of the rotation direction and communicates with the water inlet at the front of the rotation direction, resulting in small fluctuations in the outlet pressure.

### 6.4. Observation of Flow Field in the TRP

**Figure 16:**
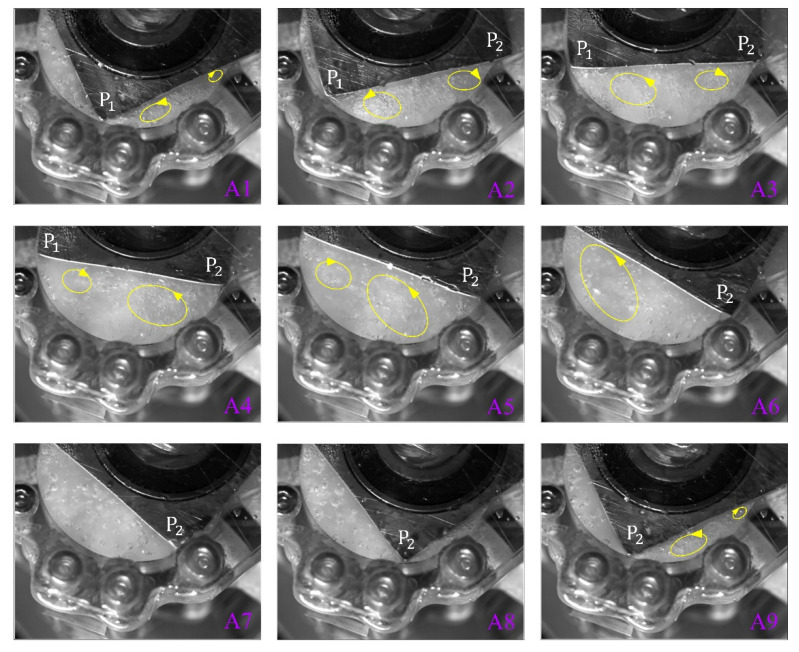
Vortices captured by a high-speed camera (Yellow circles represent vortices, and the arrow represents the direction of the vortices). (**A1**–**A9**) The actual movement of the fluid in the process of taking over the initial position of the rotor apex ($P_{1}$) by the next apex ($P_{2}$ ) when the rotating shaft is moving in a clockwise direction.

Because there is air in the pump cavity before operation, once the pump starts, the air cannot be discharged, so the pump cavity retains a small amount of air, and when the pump sucks in liquid, the pump cavity is filled with liquid and a small amount of air. Driven by the motion of the rotor, the air floats in the chamber in the form of bubbles. Figure 16 shows the bubble movement track captured by a high-speed camera at a speed of 400 frames per second. It is obvious that there are vortices in the chamber. Figure 16(A1–A9) represent the actual movement of the fluid in the process of taking over the initial position of the rotor apex ($P_{1}$) by the next apex ($P_{2}$) when the rotating shaft is moving in a clockwise direction. The yellow circles marked in Figure 16 represent vortices, and the arrow represents the direction of the vortices. As shown in Figure 16(A1), the inlet fluid is ejected into the chamber, and part of the fluid forms a counterclockwise vortex along the direction of rotation of the rotor, and another part of the fluid flows in the opposite direction of rotation of the rotor, forming a small clockwise vortex at the rear. As the chamber corresponding to rotor surface $P_{1}P_{2}$ continues to expand, the vortices gradually expand, as shown in Figure 16(A2,A3). When the apex ($P_{1}$) turns past the outlet, a small clockwise vortex appears near the outlet, which may be caused by the liquid in the chamber corresponding to $P_{1}P_{4}$ flowing along rotor surface ($P_{1}P_{2}$) from the gap at the apex ($P_{1}$), whereas the inlet continues to absorb liquid and move along the rotating direction of the rotor, forming a counterclockwise vortex near the entrance, as shown in Figure 16(A4). As shown in Figure 16(A5), the volume of the chamber corresponding to $P_{1}P_{2}$ reaches its maximum, and the vortices gradually expand. As shown in Figure 16(A6–A8), the volume of the chamber corresponding to $P_{1}P_{2}$ begins to decrease, and the fluid in the chamber flows out through the outlet. The movement of the vortex can still be seen in Figure 16(A6–A8) depicts the discharge process with no vortex current. The direction of fluid flow is divided into two parts: one part flows out through the outlet, and the other flows backward along the gap at apex $P_{2}$. As shown in Figure 16(A9), apex P_2_ takes the place of apex $P_{1}$ in Figure 16(A1) and repeats the cycle. Flow field observation experiments revealed that there were abundant vortices in the chamber, which indicates that the TRP may function as a mixer, and the design of its three suction ports and three discharge ports may be beneficial to increase the input variety, thereby increasing the mixing function of the pump.

## 7. Conclusions

In this paper, we introduced a design method and basic working principle of a new type of tetragonal rotor pump. The present study represents the first introduction of a tetragonal rotor pump based on mathematical description and theoretical analysis. The results are as follow:

(1) First, the closed equation of the chamber profile of the chamber was derived, which can be described by mathematically creating an epicycloid. Then, the closed equation of the rotor profile was derived. The rotor profile is the conjugate envelope of the chamber profile.
(2) The chamber profile and rotor profile can be determined by the eccentric distance (*e*) and the generating radius (*R*), and their ratio is defined as the shape factor (*K*). According to the industry-standard *K*-value range of 6~8, in this study, we analyzed the influence of four groups with different shape factors *K* (5, 6, 7, and 8) on the kinematic characteristics, theoretical flow rate, and specific flow rate of the proposed tetragonal rotor pump.
(3) We deduced the equations of rotor apex velocity and acceleration, which with the shape factor (*K*). Analysis of the influence of *K* on the kinematic characteristics shows that the higher the *K* value, the lower the fluctuation of the rotor apex velocity and the smaller the apex acceleration, which is conducive to reducing the interface wear between the rotor and the chamber.
(4) We also deduced the equation of the theoretical flow rate, which changed with *K*. Because the difference between the maximum chamber area and the minimum chamber area ($F_{max}-F_{min}$) decreases as the value of *K* increases, the higher the *K* value, the lower the theoretical flow rate of the pump. Furthermore, we deduced the equation for the variation in specific flow rate relative to the shape factor (*K*). Our analysis shows that the lower the *K* value, the higher the specific flow, which means that the rotor per unit volume can pump more fluid, and the overall structure of the pump is more compact. We also derived the flow rate irregularity of a tetragonal rotor pump. Compared with existing rotary positive-displacement pumps (bicornous rotor pumps and triangular rotor pumps) based on the Wankel engine structure, the TRP has lower flow rate irregularity.

As part of this study, four groups of pumps with different shape factors were manufactured, and their flow and pressure characteristics were experimentally investigated to characterize the pump performance. The inherent mixing ability of the proposed design was also illustrated. In future work, the proposed design will be optimized to achieve improved output performance.

## Figures and Tables

**Table 1:** Parameter values (unit: mm).

	Eccentricity (*e*)	Generating Radius ^®^
Pump A (*K* = 5)	5.6	28
Pump B (*K* = 6)	4.666	28
Pump C (*K* = 7)	4	28
Pump D (*K* = 8)	3.5	28

**Table 2:** Structural parameters of the pump.

	Thickness of the Rotor	Intake/Discharge Port Size	Distance between the Intake Port and Discharge Port
Pump A (*K* = 5)	18 mm	Φ6 mm	11 mm
Pump B (*K* = 6)	18 mm	Φ6 mm	11 mm
Pump C (*K* = 7)	18 mm	Φ6 mm	11 mm
Pump D (*K* = 8)	18 mm	Φ6 mm	11 mm
